# Metalorganic chemical vapor deposition of InN quantum dots and nanostructures

**DOI:** 10.1038/s41377-021-00593-8

**Published:** 2021-07-20

**Authors:** Caroline E. Reilly, Stacia Keller, Shuji Nakamura, Steven P. DenBaars

**Affiliations:** 1 Materials, University of California, Santa Barbara, CA 93106 USA; 2 Electrical and Computer Engineering, University of California, Santa Barbara, CA 93106 USA

**Keywords:** Quantum dots, Lasers, LEDs and light sources

## Abstract

Using one material system from the near infrared into the ultraviolet is an attractive goal, and may be achieved with (In,Al,Ga)N. This III-N material system, famous for enabling blue and white solid-state lighting, has been pushing towards longer wavelengths in more recent years. With a bandgap of about 0.7 eV, InN can emit light in the near infrared, potentially overlapping with the part of the electromagnetic spectrum currently dominated by III-As and III-P technology. As has been the case in these other III–V material systems, nanostructures such as quantum dots and quantum dashes provide additional benefits towards optoelectronic devices. In the case of InN, these nanostructures have been in the development stage for some time, with more recent developments allowing for InN quantum dots and dashes to be incorporated into larger device structures. This review will detail the current state of metalorganic chemical vapor deposition of InN nanostructures, focusing on how precursor choices, crystallographic orientation, and other growth parameters affect the deposition. The optical properties of InN nanostructures will also be assessed, with an eye towards the fabrication of optoelectronic devices such as light-emitting diodes, laser diodes, and photodetectors.

## Abstract (web summary)

This review article describes the current state of InN nanostructure and quantum dot growth by metalorganic chemical vapor deposition and the use in optoelectronic applications.

## Graphical abstract

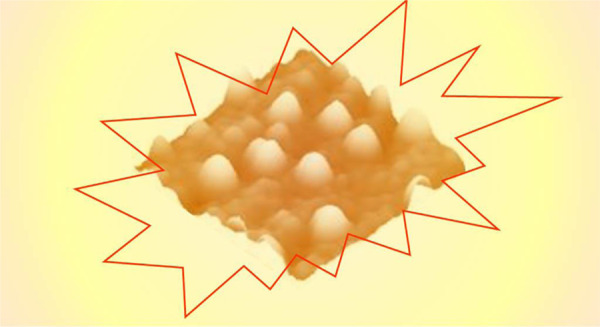

## Introduction

Nitrides devices have been a significant area of research in the past 30 years since the invention of the blue light-emitting diode (LED); however, most of the work has been in the visible or ultraviolet wavelength regime. The III-N system has been overshadowed in the infrared (IR) wavelength device area by other III–V semiconductors such as arsenides and phosphides^1–3^. Although these other material systems have seen significant success in IR devices, challenges remain when operating devices at elevated temperatures^4,5^. For instance, a small band offset between the active region material and the cladding material in laser diodes can lead to carrier loss to the cladding material at higher temperatures^6–8^. Using InN, with a bandgap of about 0.7 eV, the nitrides can overcome these challenges and allow the fabrication of IR devices for higher temperature operation^9^. The InN/GaN system has large band offsets, resulting in better confinement of carriers in the active regions due to the wide bandgap of GaN^10^.

**Fig. 1:**
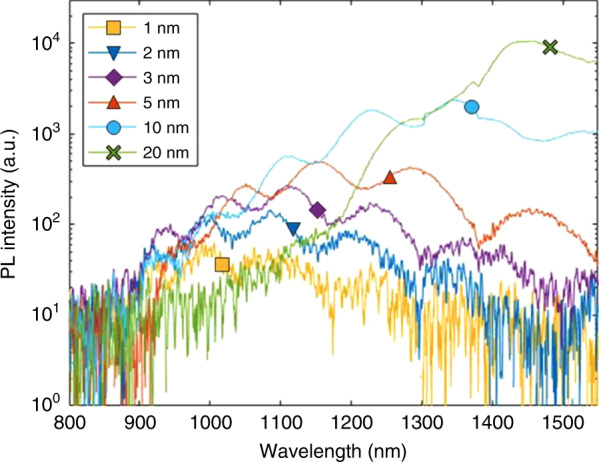
Room temperature PL from N-polar InN QDs grown by MOCVD with varying nominal thicknesses. Increasing intensity and red-shifted emission seen as nominal thickness increases. Reprinted from Reilly et al., *Appl. Phys. Lett.* **114**, 241103 (2019) with permission of AIP Publishing^78^

InN quantum dots/dashes (QDs) and nanostructures are at the forefront of nitride-based IR research. Three-dimensional structures are promising over planar structures for a wide variety of applications from quantum computing and single-photon emission to laser diodes and LEDs^11–13^. Benefits derive from the quantum confinement in multiple dimensions and carrier confinement in-plane. The zero-dimensional density of states in QDs can allow for extremely low full width at half maximum (FWHM) emission from a single QD, with the emission wavelength dictated by the size of the QD, such as in the photoluminescence (PL) data in Fig. 1. By precisely controlling the size of the QDs, a very sharp linewidth emission can be achieved. In addition to quantum confinement controlling the emission energy by blue-shifting the emission with reduced sizes, carrier confinement in QD structures used as device active regions can improve efficiency in several ways. As compared to planar quantum well (QW) structures, QD layers can be less sensitive to threading dislocations which act as non-radiative recombination centers^14,15^. If the QD growth sites are not inherently correlated with the threading dislocations, any given QD would have some random chance to be placed at a threading dislocation site. A QW, on the other hand, would interact with nearly every possible threading dislocation due to covering the entire surface. Additional benefits exist when using QDs in lasers, where less temperature sensitivity and reduced threshold current are possible^16,17^.

In the case of InN, another significant reason to use nanostructures over planar layers is due to strain and lattice mismatch considerations. The lattice mismatch between InN and GaN is over 10%, leading to very small critical thicknesses of under 1 ML when InN is grown coherently on GaN. Studies have shown that surface reconstruction leads to the formation of InGaN when sub-monolayer thick layers of InN are deposited on GaN^18,19^. Some reports of monolayer thick InN have been reported which may be used for digital alloy applications^20–22^; however, these very thin layers are not suited for active regions if IR emission is desired^23^. Thick planar layers of InN have also been reported, which may be interesting for electronic applications but have limited use in optoelectronics, where relatively thin layers are desirable for device active regions^24,25^. InN QDs typically start to form under one monolayer of thickness due to the high strain in the InN/GaN system and are good candidates for device active regions^26,27^. The natural formation of QDs when growing under high lattice mismatch conditions can be leveraged for InN QD devices.

The two main candidates for the epitaxial growth of InN QDs are molecular beam epitaxy (MBE) and metalorganic chemical vapor deposition (MOCVD)—the standards for semiconductor epitaxy. Significant work on InN QDs and nanostructures has been conducted via MBE^28–32^, which has helped to direct the MOCVD studies. MOCVD is typically used to grow nitride LEDs and lasers commercially, due to faster growth rates and the ability to scale up the process. In addition, as MOCVD is typically used to grow the GaN templates and other device layers, such that growing the InN QDs by MOCVD would prevent the need to use multiple growth techniques for a single device stack. In addition to the two main growth techniques, epitaxial InN nanostructures have also been grown by various other methods including plasma-assisted MOCVD^33^, gold-assisted nanowire growth^34,35^, plasma aerotaxy^36^, oblique angle deposition method^37^, chemical beam epitaxy^38^, and chemical vapor deposition^39–43^. Although MOCVD has the potential to be the preferred growth option for InN QDs due to scalability and fast growth rates, the high growth temperatures (>1000 °C) typical for high-quality GaN growth pose challenges for the incorporation of InN into device stacks. InN is typically grown below 700 °C and decomposes at higher temperatures, necessitating low temperature (LT) growth schemes for both InN and GaN grown on top of the QDs. Raising the growth temperature after the growth of InN can also cause intermixing of the InN and the GaN. Studies on capping the InN QDs, including LT growth schemes, are then critical to the ability to create InN QD devices.

**Fig. 2:**
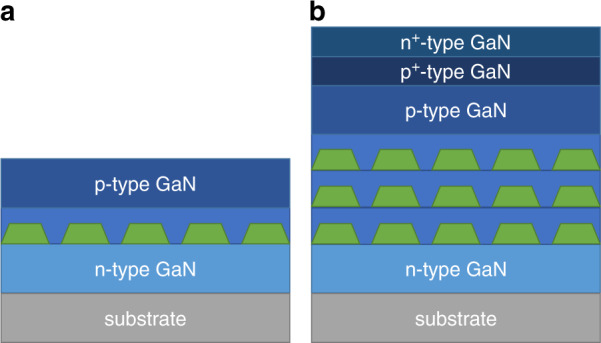
Schematics of simplified device structures featuring InN QD active regions. A structure with a single layer of InN QDs is shown in (**a**) and a structure with three layers of InN QDs with a tunnel junction is shown in (**b**).

This review will focus on the MOCVD growth of self-assembled InN QDs and nanostructures on GaN, with a specific emphasis on the use of these structures in optoelectronic devices such as LEDs, laser diodes, and photodetectors. The effect of metalorganic precursor choice will be considered along with typical growth parameters such as temperature, growth rate, V/III ratio, and nominal thickness. Incorporating InN QDs into full device structures (Fig. 2) through various capping schemes will be explored, including recent advances in LT GaN growth. The shape, size, and density of the QDs will be analyzed across the reports in the literature for different crystallographic orientations. With very few device results demonstrated to date with this material system and no reports on electroluminescence, this article will focus on the reported material characterization and PL results. Optical properties of the InN QDs will be discussed and gaps towards the success of IR nitride optoelectronics will be addressed.

## LT growth

### Growth parameters

The range of QD applications necessitates that dot parameters such as shape, width, height, and density can be tuned to match the application. Choices such as which crystal plane to grow on or what metalorganic precursors to use can greatly affect the framework of the growth and affect properties such as the shape of the nanostructures. Other parameters including growth rate, V/III ratio, growth temperature, and nominal thickness can additionally be utilized to further tune aspects of the QDs. Some of these parameters have trends that can be expected irrespective of orientation or growth rate choices, whereas others may depend more on the specific growth conditions. In general, increased growth rates can be expected to increase density and decrease the size of the nanostructures. V/III ratio needs to be sufficiently high for a given temperature in order to achieve any InN growth, where low V/III ratios can lead to In droplet formation^44^. The effects of temperature can be broken down by considering the effects of temperature on surface adatom mobility and growth rate. Higher temperatures will increase surface adatom mobility; however, the effect of temperature on the growth rate depends on the choice of group III precursor, as will be discussed in the next section. Decreasing surface adatom mobility can have a similar effect as increasing growth rate.

**Fig. 3:**
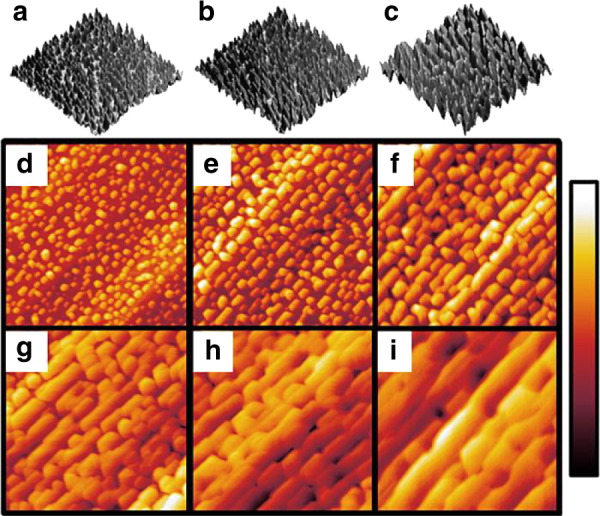
Atomic force micrographs of InN on N-polar GaN. Nominal InN thicknesses of (**a**)/(**d**) 1 nm, (**b**)/(**e**) 2 nm, (**c**)/(**f**) 3 nm, (**g**) 5 nm, (**h**) 10 nm, and (**i**) 20 nm. **a**–**c** Show 3D images whereas (**d**)–(**i**) depict top down images with the scale at the right as follows: −5 to 5 nm for (**d**)–(**f**) and −10 to 10 nm for (**g**)–(**i**). Reprinted from Reilly et al., *Appl. Phys. Lett.* **114**, 241103 (2019) with permission of AIP Publishing^78^. Adapted from Lund et al., *J. Appl. Phys.* **123**, 055702 (2018) with permission of AIP Publishing^48^

Another main tuning parameter is the nominal thickness or the amount of material deposited, given as an extrapolated layer thickness if the material were to be deposited in a planar fashion. Nominal thickness can be assumed based on a calibrated growth rate or it can be calculated based on the geometry and density of the deposited QDs. The expected and calculated nominal thickness values generally correspond at longer growth times, where calculated nominal thickness and growth time are linearly related^26^. In the initial stages of growth, these values may not be directly correlated if a wetting layer forms^27^. Depositing more material can increase dot density and/or increase dot size, depending on the specific growth conditions. In Fig. 3, atomic force microscopy (AFM) images show the increase in size with increasing nominal thickness of InN QDs grown on N-polar GaN.

### Group III precursors

LT MOCVD growth presents challenges associated with reduced decomposition efficiency of metalorganic precursors as well as increased impurity incorporation^45^. At reduced temperatures, the decomposition of metalorganic precursors occurs at a lower rate, such that some metal-C bonds may still be intact while incorporating the metal into the film. These concerns have driven some studies to turn to alternative precursor studies for both InN and GaN^46^. For GaN growth, triethylgallium (TEGa) is often used as a group III precursor for layers grown at lower temperatures or where low impurity incorporation is especially important. The other standard Ga precursor is trimethylgallium (TMGa), in which the Ga-C bonds are less easy to break and therefore more carbon tends to incorporate into the films. TEGa tends to incorporate less carbon, despite having more carbons per Ga, due to the beta-hydride elimination process which forms ethene and extracts the carbon more efficiently^47^. In a similar fashion, the less utilized triethylindium (TEIn) may serve to incorporate less carbon than the commonly used trimethylindium (TMIn) while growing at very low temperatures.

The majority of InN QD growth to date has been conducted with TMIn^26,44,48–53^. A few studies have been conducted on InN QDs which were grown using TEIn^27,54^. One group showed that TEIn could be used to grow thick layers of InN, where the growth rate by TEIn was twice that as by TMIn with the same amount of precursor injected^46^. The growth rate for these precursors is expected to be a function of temperature with the high-temperature growth limited by the desorption of InN and the LT growth limited by the precursor decomposition. For the low temperatures at which InN is typically grown, the group V precursor deposition may also limit the LT growth rate. As TEIn and TMIn have different decomposition rates, the difference in TEIn and TMIn growth rates found in the study is not surprising and associated with the higher decomposition efficiency of TEIn at that growth temperature. While this is another reason to consider TEIn for LT MOCVD, the higher cost and lower availability of TEIn, in addition to the low vapor pressure, has led to it being less used to date.

### Group V precursors

The primary N precursor used in MOCVD is ammonia, which is supplied in the gas form in large quantities in order to achieve high V/III precursor ratios. The decomposition rate of ammonia decreases at lower temperatures, leading to concerns about insufficient available nitrogen during LT MOCVD growth. In particular, the growth of InN necessitates a higher V/III ratio than that of GaN or AlN due in part to the difference in equilibrium partial pressures of nitrogen^55^. The decomposition of ammonia via surface catalysis has been seen to be sufficiently large for most LT growth regimes; however, there has been interest in alternative N precursors such as dimethylhydrazine (DMHy) and tertiarybutylhydrazine (TBHy) as well^56–59^. Arsine- and phosphine-based counterparts of these precursors have seen usage in III-As/P MOCVD growth. The benefit of these alternative precursors would be significant if the amount of total active nitrogen could be greater in the case of using DMHy or TBHy instead of ammonia. As DMHy and TBHy are relatively low vapor pressure metalorganics, even when fully decomposed the low vapor pressures of DMHy and TBHy create challenges in providing enough active nitrogen. While the growth mechanism may additionally change with the use of these alternative N precursors to somehow provide additional benefits to the material grown, the minimum amount of nitrogen still needs to be provided.

Due to the temperature-dependent decomposition rates of the N precursors, the V/III ratio can vary with the growth temperature. At higher temperatures, less ammonia was necessary to achieve InN growth^44^. At lower temperatures, if the ammonia flow did not reach a minimum amount, In droplet formation was seen without growth of InN. In droplet formation was also seen for attempted growth of InN using both DHMy and TBHy, potentially due to the low V/III ratios which can be established with these precursors because of their low vapor pressures^46^. However, the LT growth of GaN has proven successful using both DMHy and TBHy, in some cases showing enhancement in material quality in comparison to films grown using ammonia at similar temperatures^46,57,59^.

### Pulsed versus continuous growth

Another proposed way to mitigate the problems associated with LT growth is through the use of pulsed growth schemes such as flow-modulation epitaxy (FME)^60–63^. In this growth scheme, one or both of the precursors are pulsed to allow adatoms more time to move about the surface. Modulating the precursors in this fashion can help to combat the issues of low adatom diffusion distances which occur due to reduced surface adatom mobility during LT growth. This generally results in very slow growth rates and has the potential to improve morphology and reduce impurities compared to continuously grown LT films. With this technique, more parameters are available to tune the layers including the precursor pulse shapes and duty cycles. The use of this method can be applied to both the InN QDs themselves as well as the LT GaN capping material. One study on the FME growth of InN QDs found that the dot shape and density could be controlled by optimizing the background ammonia level between pulses of ammonia^64^. This provides another way to tailor the dot conditions and the study resulted in brighter PL from the InN QDs.

When considering growth schemes for LT GaN cap growth there are two concerns, the morphology of the GaN and the impurity incorporation. Achieving reasonably low roughness films with low impurity levels can lead to better electronic characteristics of films. In particular, n-type or p-type layers require low residual impurity incorporation to prevent charge compensation effects in the films. In addition to wanting a low surface roughness, the morphology of GaN caps would ideally planarize the surface such that the QD layer can be stacked and further device layers above the QDs are relatively flat for growth and processing considerations. The step-flow growth of smooth GaN layers has been shown via FME at temperatures useful for application as InN QD cap layers for metal-polar growth^65^. By varying parameters such as injected precursor flow and the on-time of the pulses, films with visible steps and roughness on the order of that of high-temperature GaN were achieved for thin layers. In addition, the carbon and oxygen impurity incorporation was shown to be significantly reduced compared to continuous growth methods^65^.

**Fig. 4:**
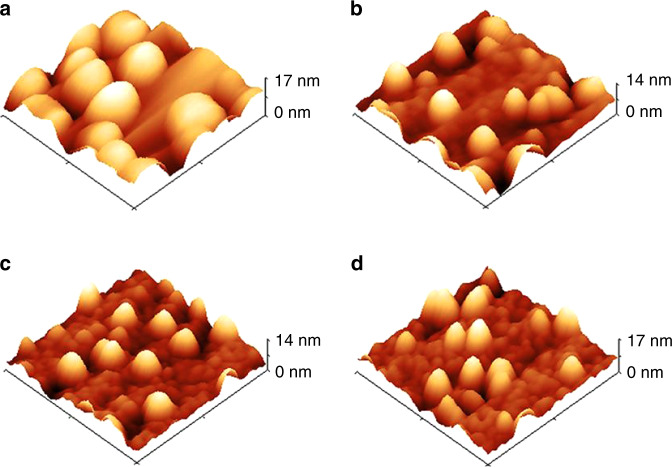
InN QDs in 3D AFM (300 nm)^2^ scans. Continuously grown GaN cap thicknesses of (**a**) 0, (**b**) 12, (**c**) 18, and (**d**) 23 nm. From Reilly et al., *Phys. Status Solidi Basic Res.* **257**, 1900508 (2020) © 2019 WILEY-VCH Verlag GmbH & Co. KGaA, Weinheim^54^

FME GaN films were used to cap InN QDs, and the X-ray diffraction (XRD) signal from the InN remained mostly unshifted compared to the expected position of InN, suggesting little to no Ga incorporation into the InN QDs^65^. This effect was not necessarily attributed to the FME growth; however, as InN QDs were also capped via continuously grown GaN, with similar XRD results^54^. Further reports indicated via atom probe tomography (APT) that the method of continuously growing the GaN cap maintained the composition of the InN QDs^27^. Unfortunately, AFM images showed that the continuously grown films were conformal and unsuccessful in planarizing the metal-polar InN QDs after cap growth (Fig. 4)^54^. Thus, dots with heights less than the thickness of the GaN cap layers were visible after capping. Comparatively, in the case of N-polar InN QDs capped with FME GaN, TEM images showed that QDs were planarized after GaN growth^48^. However, the metal-polar and N-polar QDs were significantly different in shape and size, suggesting that the crystallographic nature of the dots strongly affects their ability to be planarized. Thus, the effect of crystal orientation on QD properties will be discussed in the next section.

## Crystal orientations

### Metal-polar or (0001)

The most common growth plane for III-nitride optoelectronics is the (0001) plane, also known as the *c*-plane or the metal-polar orientation. InN QDs are no exception, with the majority of studies being on metal-polar GaN^26,27,49–52,54^. In this orientation, spontaneous and piezoelectric polarization typically have strong effects which depend on the composition and strain state of the material^66^. In the case of InGaN QWs coherently grown on GaN, the polarization leads to the quantum-confined Stark effect (QCSE), which reduces the radiative efficiency and red-shifts the emission wavelength. As the QCSE increases with increasing In content, one would expect InN QDs to be highly affected by this. However, the redistribution of strain in QDs, compared to QWs, can lead to a reduction in the QCSE^67^. Additionally, any relaxation of the InN will remove the piezoelectric component of the polarization such that the QCSE may not play a significant role in the operation of relaxed metal-polar InN QDs, as spontaneous polarization only plays a subordinate role in the (In,Ga)N system. The metal-polar orientation is then a reasonable candidate for InN QD devices, in particular due to the wealth of other growth studies on this plane.

**Fig. 5:**
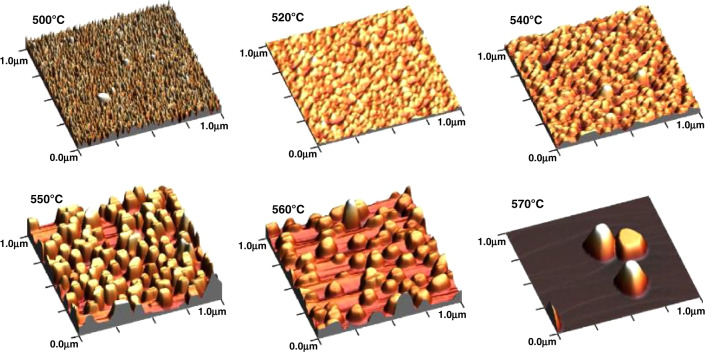
InN QDs in 3D AFM (1 μm)2 scans with indicated growth temperatures. Reprinted from Meissner et al., “Indium nitride quantum dot growth modes in metalorganic vapor phase epitaxy”. *J. Cryst. Growth*, 310/23, 4959-4962, Copyright (2008), with permission from Elsevier^51^

Metal-polar InN QDs typically have a truncated hexagonal pyramid shape with sidewall angles indicating a specific semi-polar plane, seen in some cases to be 60° and corresponding to {10−11} sidewalls^27^. The aspect ratios of the dots vary across different reports and growth conditions but are typically in the range of width-to-height ratios of 2–10^44,54^. Likewise, QD density, height, and width vary with specific growth conditions across the literature; however, trends hold for most reports. Typical values for height, width, and density are on the order of 5–50 nm, 20–200 nm, and 10^7^–10^11^ cm^−2^, respectively. At very low growth times, no QD growth occurs, corresponding to the growth of an incomplete wetting layer^27^. During the initial nucleation of the dots, more material deposition corresponds with sharply increasing dot densities which then level off as the dots increase in size^26,27,54^. Increasing growth times leads to larger dots, in both height and width, up until a point where diameter stops increasing and the dots continue to grow taller^26,54^. Growth temperature significantly impacts both dot size and density, as can be seen in Fig. 5 for InN QDs grown with TMIn^51^. Within the temperature ranges reported and for both TMIn and TEIn, increasing temperature leads to decreased density and increased size, with large changes occurring over a range of 100 °C^44,51,52,54^.

### N-polar or (000$\bar 1$)

The N-polar orientation is also referred to as −c-plane or $(000\bar 1)$ plane and has been less well studied as a growth plane for InN QDs and for III-nitrides in general^68^. Applications of the N-polar plane are largely in III-N electronics^69–72^. N-polar InGaN has been reported to show less efficient luminescence than metal-polar InGaN in the low In content range and is therefore less attractive for blue and green optoelectronic applications^73,74^. However, InN has unique properties compared to the other nitrides by virtue of its small bandgap, such that some of the defect levels which lie within the bandgap in GaN, InGaN, or AlGaN are within the conduction band in InN^10^. Additionally, surface layers of polar InN can show luminesce where low In content InGaN cannot, due to charge buildup at the surface of InN because of surface pinning^75–77^. While the differences in luminescence between the metal- and N-polar planes are not necessarily associated with these properties, the behavior of InN is unique enough such that the luminesce differences should be reevaluated in the InN system. In the N-polar orientation, being the reverse of the metal-polar orientation, the internal electric fields caused by the spontaneous and piezoelectric polarization are reversed as well. In contrast to the Ga-polar plane, the N-polar plane is a very slow-growing plane when coexisting with other available planes, explaining the variations in QD shape and size mentioned previously.

In reports of N-polar InN QDs by MOCVD, vicinal GaN-on-sapphire with a 4° miscut towards the GaN m-direction was used as the growth substrate^48^. Rather than a dot-like structure, quantum dashes were observed with alignment along the terraces in the underlying GaN. By TEM, these QDs were seen to have a wedge profile with a −c top surface and an inclined sidewall^48^. After nucleation, increasing the nominal thickness led to the growth of the QDs along the terraces until the point of film coalescence around nominal thicknesses of 5–10 nm at 640 °C (Fig. 3)^78^. Decreasing the growth temperature causes QDs height to decrease with an increase in length along the terraces. Unlike the metal-polar QDs, the N-polar dashes remain relatively short in height for a range of growth conditions. The higher lateral growth rate compared to the vertical growth rate also allows for planarization of the N-polar QDs with a GaN cap and enables the growth of stacked QD layers.

### Other orientations

Other planes in the III-N material system include non-polar and semi-polar planes. Non-polar planes such as the a- and m-planes are perpendicular to the *c*-plane, with no polarization. Semi-polar planes are all other planes which create some angle with the *c*-plane other than 90°. These planes have polarizations which are less than that of the c-plane but are still nonzero, allowing for polarization engineering depending on the application desired^79^. Typically, GaN growth on these planes shows surface undulations. This template morphology may affect the QD growth shape and size on these surfaces, as it has been seen in the N-polar case. Some work by MOCVD has shown thick planar layers of InN grown in the non-polar and semi-polar orientations, on GaN-on-sapphire or sapphire directly^80,81^. In another study, the growth of InN QDs on freestanding $\left( {20\bar 2\bar 1} \right)$ GaN resulted in long dashes with a triangular side profile, aligning along the a-direction with the striations in the underlying GaN^82^. With increasing growth time, these InN QDs grew in both length and height, eventually overlapping at long growth times.

**Fig. 6:**
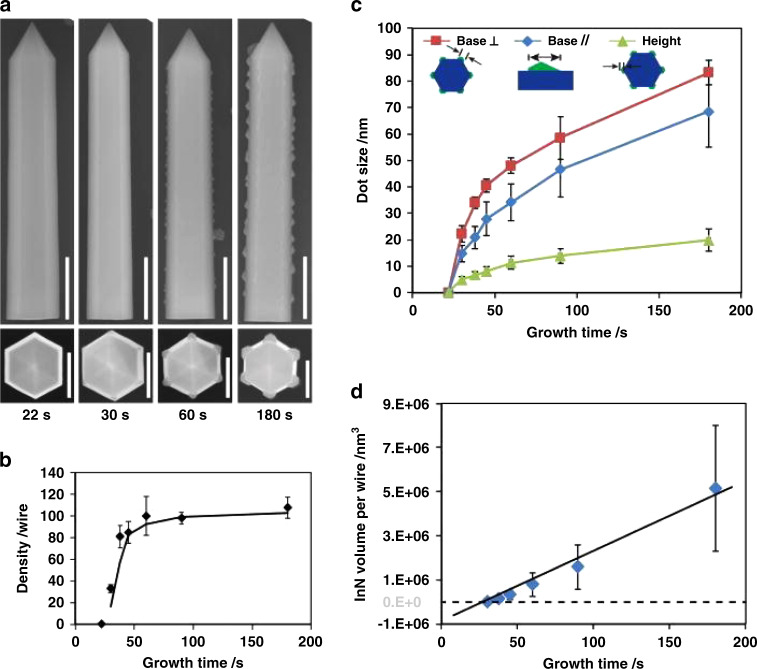
InN QDs on GaN NWs at varying growth times. **a** SEM images of side (top) view with 500 nm scale bar and top (bottom) view with 250 nm scale bar. Plots of InN QD parameters versus growth time: (**b**) density per wire, (**c**) size, and (**d**) InN volume on each nanowire. Reprinted from Bi et al., *J. Appl. Phys.* **123**, 164302 (2018), with the permission of AIP Publishing^53^

While non-polar and semi-polar GaN can be expensive due to the difficulty in achieving bulk GaN templates, these crystallographic orientations can also be accessed through the use of GaN nanostructures such as nanowires, as reported by Bi et al.^53^. InN QDs were nucleated on the side facets and edges of GaN nanowires, where the side facets of the nanowires corresponded to the non-polar m-plane^53^. Faceting was visible on the InN QDs corresponding to various crystal planes with asymmetric dots showing a −c-plane facet, as indicated in the insets in Fig. 6b. The preferential nucleation of the InN QDs was on the edges of the nanowires, as seen in Fig. 6a. QDs also grew on the sides of the nanowires at increased growth rates and decreased temperatures, due to the lowered diffusion length of surface adatoms in that regime^53^. A wetting layer was noted in the growth of these InN QDs, leading to no InN QD growth at very low growth times. Increasing nominal thickness, corresponding to increasing growth time in Fig. 6b–d, lead to an initial sharp increase in density which then leveled off at longer growth times. The dots size continued to increase in all three reported dimensions, and the calculated InN QD volume corresponded approximately linearly with growth time past the wetting layer formation^53^.

## Optical properties/devices

### Photoluminescence

With the end goal of creating optoelectronic devices in mind, the emitting properties of InN QDs are critical to their overall adoption as a technology. Typically, efficient PL is a first step towards efficient electroluminescence (EL), where efficient EL in LEDs is then desirable before considering the use of a material for lasers. PL can be measured from incomplete devices without the need for cap layers for InN. The effects that the previously discussed InN QD parameters have on the PL properties can then be a proxy for their EL properties. PL signals from InN QDs have been reported in the range of about 0.7–1.2 eV^50,52–54,64,78,82,83^. The emission energy for any given QD is controlled primarily by the bandgap of InN as well as the size of the QD. The smallest dimension, typically the height in the case of InN QDs, dictates the shift in emission from the bandgap. Blue-shifts in the PL have been seen with decreasing size QDs across multiple studies on different orientations, including all those discussed at length above (Fig. 1)^52–54,78^. Other effects such as the Burstein–Moss effect can also contribute to the emission energy and intensity, as seen in the case of InN films with high carrier densities^78,84,85^.

In addition to the emission wavelength being important, the FWHM of the emission spectrum can also be relevant for many applications. As previously mentioned, for a fully 3D confined system such as a single QD, the zero-dimensional density of states should make the emission a delta function with a very sharp emission spectrum. As the specific energy for each QD is controlled by the QD size, the emission FWHM of an array of QDs is controlled by the size distribution of the QDs. This can lead to relatively broad emission in some cases, although an extensive study has not been reported. Another area in which more work is needed is a thorough analysis of the quantum yields and emission efficiencies for MOCVD grown InN QDs. Reports of a Volmer–Weber growth mode along with relaxation and misfit dislocations formed at the GaN/InN interface^26,49,86^, in conjunction with the majority of the literature showing only LT PL^50,53,64,82^, have indicated that this is an area of concern. However, several more recent reports of room temperature PL suggest the possibility of higher emission efficiencies^54,78,83^. One report showed a rather modest PL intensity decrease of 20% between 80 K and 300 K^52^; however, further studies are needed to determine the emission efficiencies of InN QD structures.

**Fig. 7:**
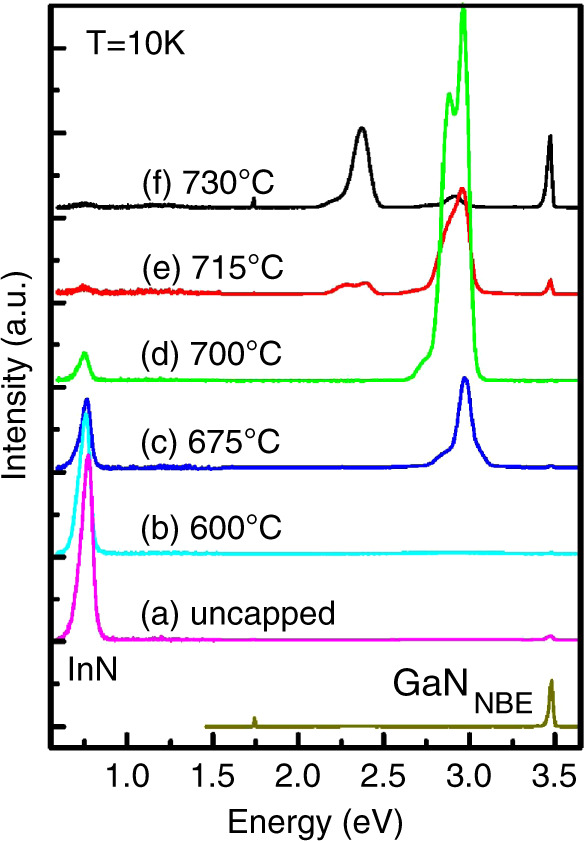
Micro-PL at 10 K from InN QDs capped with GaN at varying GaN growth temperatures. Reprinted from Ku et al., *Appl. Phys. Lett.* **90**, 132116 (2007), with the permission of AIP Publishing^50^

**Fig. 8:**
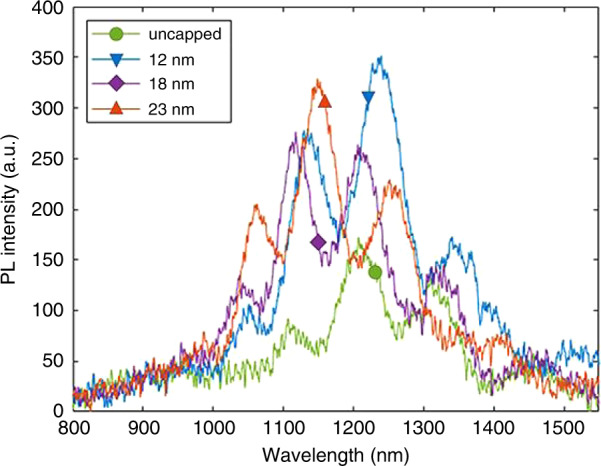
PL from InN QDs capped with varying thicknesses of GaN. From Reilly et al., *Phys. Status Solidi Basic Res.* **257**, 1900508 (2020) © 2019 WILEY-VCH Verlag GmbH & Co. KGaA, Weinheim^54^

Many of the PL studies on InN QDs have been on uncapped dots, where the surface pinning of InN and the subsequent electron accumulation at the surface can contribute to increased luminescence^75–77^. Capping the InN QDs with GaN has proven challenging, specifically to be able to maintain high PL signals. In one study, InN QDs were capped with GaN at varying growth temperatures^50^. The dots were seen to reduce in height with increasing cap growth temperature, as well as modestly decreasing in density. In addition, PL was measured from uncapped and capped InN QDs, as seen in Fig. 7. The InN PL signal decreased in intensity as the cap temperature was increased, eventually almost entirely going away and being replaced with a visible PL signal attributed to InGaN^50^. Two more recent studies have shown the ability to cap InN QDs with GaN while largely maintaining the intensity and wavelength of the PL signal^54,78^. In one study, the InN QDs were metal-polar and the GaN cap was grown continuously at 550 °C, in an atmospheric pressure MOCVD reactor^54^. As shown in Fig. 8, a slight blue shift appeared to occur after capping and the intensity of the PL was maintained or even increased. An N-polar study showed the stacking of multiple layers of InN QDs with GaN barriers in between, such that PL was maintained after capping and a sample with three layers of QDs showed increased PL over a sample with one layer of QDs^48,78^.

### Photodetectors

**Fig. 9:**
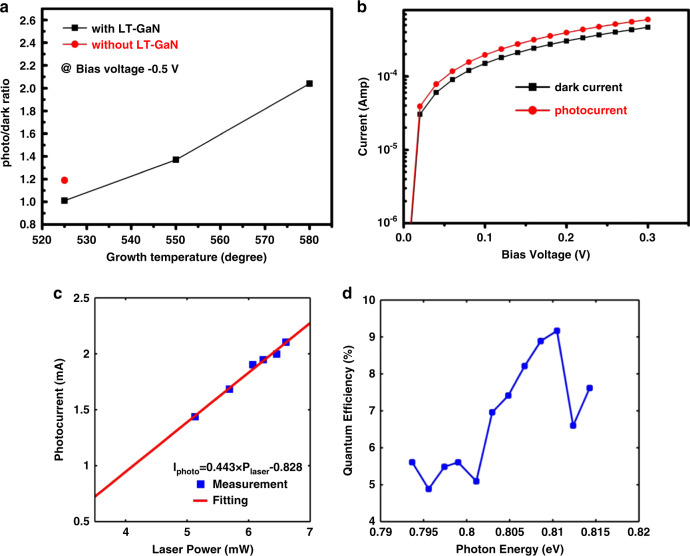
Photodetector behavior for InN QD-based Schottky photodiode. **a** Photocurrent to dark current ratio at varied InN growth temperatures, uncapped and capped. **b** Reverse bias *I*–*V* characteristics, with InN QDs grown at 525 C, with and without 1550 nm laser illumination. **c** Difference in photocurrent and dark current at −0.2 V, at varied laser powers. **d** External quantum efficiency versus photon energy. Reprinted with permission from^82^ © The Optical Society

Although this review has largely focused on the growth and emission properties of InN QDs, the use of this material for photodetectors is also of interest. Additionally, the ability to successfully achieve photocurrent when illuminated under reverse bias is a promising result towards achieving EL. While several studies using various growth techniques have shown results with InN photodetectors, none of these studies created a p–n junction photodiode consisting of entirely epitaxially grown nitride material^82,87–91^. One study fabricated a photodetector using MOCVD grown self-assembled InN QDs in a Schottky-like photodetector device, where the InN QDs were protected with a layer of undoped LT GaN^82^. In this study, the PL of the InN QDs was significantly lower in intensity after the GaN cap layer was deposited, and intermixing between the InN QDs and the cap was noted. However, as seen in Fig. 9, photocurrent response with 1550 nm illumination was observed. As these results were obtained for a system in which the LT GaN capping caused intermixing, improvements would likely follow if recently reported optimized GaN capping schemes were to be used.

### Electroluminescence

EL in epitaxial InN QDs, with a full p–n junction LED or laser structure, has yet to be reported either by MOCVD or MBE. Although capping of InN QDs has been successful to maintain composition and PL, studies have only shown capping with undoped layers of LT GaN^54,78^. Doped GaN with either p-type or n-type behavior would need to be deposited on top of the InN QDs in order to inject carriers into the active region (Fig. 2). MOCVD grown LT n-type or p-type GaN films have not been the subject of much published research to date. Higher impurity levels in LT GaN grown at temperatures comparable to those used for InN growth present challenges towards the deposition of doped LT GaN layers with sufficient carrier concentrations. Although the impurity levels in LT GaN films may not reach levels as low as in conventionally grown GaN layers, the LT FME GaN growth scheme presents a promising approach to lower the impurity incorporation^65^. Further studies are needed to optimize intentional doping of FME layers to demonstrate p–n junctions and EL.

The efficiency of the radiative recombination of carriers in the InN QDs is also of concern due to the presence of misfit dislocations at the InN QD/GaN interface^49,53,86^. These dislocations may act as non-radiative centers, reducing the efficiency. Current work suggests that the misfit dislocations cannot be mitigated via InN QD growth optimization. They have been seen for both *c*-plane and m-plane growth and are a result of the large lattice mismatch between InN and GaN causing relaxation of the InN QDs^49,53^. However, as defect-related non-radiative carrier recombination would also affect the PL of the InN QDs, the reports of room temperature PL suggest that EL will be possible. The efficiency of the PL and potential EL remains an open question which needs to be addressed in future work. Furthermore, as only very few studies have shown stacked layers of InN QDs, it is unknown if misfit dislocations also form at the base of InN QDs in subsequent layers. In other III–V QD systems, stacked QD layers have been used for device applications^1,14^. Moving to multiple QD layers in the active region may provide additional benefits towards efficient EL in InN QD structures as well.

## Conclusions

Challenges remain towards the achievement of EL in the MOCVD grown InN QD system; however, the material system has overcome some of the main roadblocks related to LT GaN growth and capping of InN QDs in recent years. A variety of precursor choices and growth schemes are available for the deposition of InN and GaN in order to improve morphology and reduce impurities—with the use of ammonia and FME as the most promising LT growth approach. Control over the density, size, and shape of the QDs has been shown via growth parameters such as crystallographic orientation, growth temperature, and nominal thickness. More work to determine emission efficiencies will be necessary to determine the need for suppressing non-radiative recombination at defects. Recent reports of maintained PL from capped InN QDs point towards the ability to achieve buried InN QD layers, with some preliminary work on photodetectors also laying the foundation for InN QD devices. Doping of LT grown layers remains a challenge towards enabling EL, with either p- or n-type LT GaN necessary. By narrowing in on the right combination of growth conditions and implementing LT growth schemes, MOCVD InN QDs are poised to bring the III-nitrides into the field of infrared optoelectronics.
